# Prostatic carcinoma with elevated carcinoembryonic antigen: a case report

**DOI:** 10.3389/fonc.2026.1701428

**Published:** 2026-03-09

**Authors:** Zhi-xin Xie, Ai-ming Yang, Qiang Wang

**Affiliations:** 1 Department of Clinical Medicine, Peking Union Medical College, Chinese Academy of Medical Sciences, Beijing, China; 2 Department of Gastroenterology, Peking Union Medical College Hospital, Chinese Academy of Medical Sciences, Beijing, China

**Keywords:** carcinoembryonic antigen, diagnostic markers, early diagnosis of prostatic carcinoma, prostate-specific antigen, prostatic carcinoma

## Abstract

Carcinoembryonic antigen is a glycoprotein often associated with colorectal carcinoma but can also increase in other malignancies. We reported a rare case of prostate mucinous adenocarcinoma in a 69-year-old male with elevated carcinoembryonic antigen and normal prostate-specific antigen. The patient, with a history of benign prostatic hyperplasia, was initially asymptomatic apart from an elevated carcinoembryonic antigen detected during routine examination. Comprehensive evaluations, including colonoscopy, CT and PET/CT, yielded no conclusive findings. However, a pelvic MRI later revealed an irregular prostatic mass, and a biopsy confirmed adenocarcinoma with mucinous features. Carcinoembryonic antigen positivity was further demonstrated by immunohistochemistry on the specimen. This case highlights that isolated carcinoembryonic antigen elevation, even in the absence of prostate-specific antigen elevation, may warrant consideration of prostatic carcinoma in differential diagnosis, particularly in the absence of colorectal carcinoma history. Elevated carcinoembryonic antigen levels should prompt further investigation, as they may serve as an early marker for prostatic malignancies when followed by targeted imaging and histological confirmation.

## Introduction

Carcinoembryonic antigen (CEA) is a glycoprotein normally derived from embryonic endodermal epithelium in the fetus. It was first isolated from human colorectal carcinoma (CRC) and was later found to exist in various malignant and benign diseases (1–3). The serum CEA test contributes to monitoring the therapeutic efficacy, recurrence, and metastasis of colorectal carcinoma (4). It is also widely applied in clinical practice to aid the diagnosis of CRC despite its unsatisfactory sensitivity (5). Apart from CRC, the CEA level is also clinically applied in the diagnosis of ovarian cancer and cervical cancer. In recent years, the application of CEA has been extended to the auxiliary diagnosis of other malignant diseases, including medullary thyroid carcinoma, pancreatic cancer, and hepatic carcinoma, playing a role as a clue instead of an essential diagnostic indicator. However, an elevated CEA level as the first and only change in clinical indices may bring difficulties with the diagnosis and differential diagnosis of colorectal carcinoma and other malignant diseases. In this report, we review a case of a male patient who had an elevated CEA level as the only early appearance and was finally diagnosed with prostatic carcinoma, which is rare in the clinic.

## Case description

**Figure 1:**
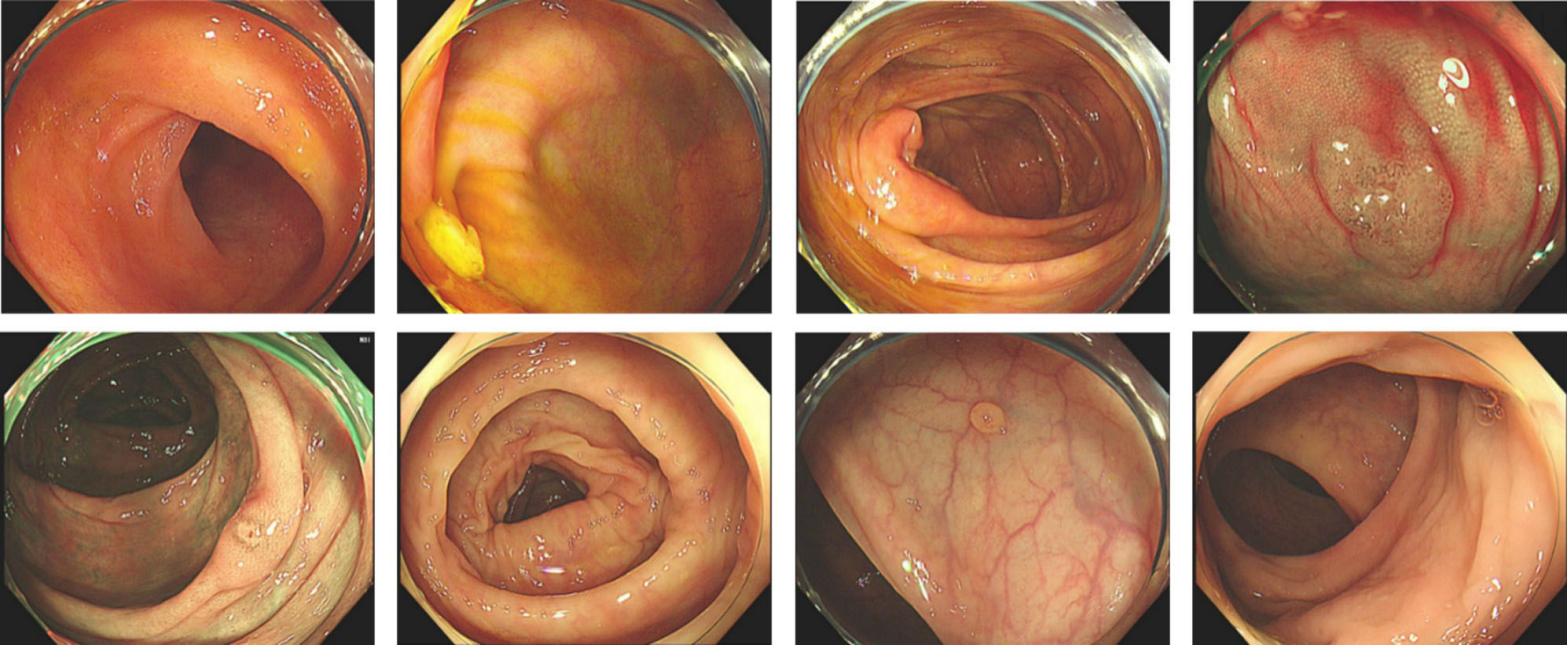
Colonoscopy findings of various colonic lesion. Colonoscopy shows a 0.6 cm basal raised lesion near the hepatic flexure, a 0.3 cm erosive lesion in the middle transverse colon, and a semipedunculated polyp in the sigmoid colon.

A 69-year-old man presented to the Gastroenterology Department in February 2023 with complaints of an elevation in CEA (7.5 ng/mL, reference range ≤ 5.00) during a physical examination over four months ago. The patient had a history of prostatic hyperplasia for over 10 years and was regularly treated with finasteride and doxazosin. Enhanced abdominopelvic computed tomography (CT) imaging revealed prostate enlargement with slight uneven enhancement and multiple calcifications. In May 2023, shortly after consultation, the patient underwent gastroscopy and colonoscopy examinations. The gastroscopy revealed red gastric antral mucosa with scattered erosion and focal hyperemia in the prepyloric region, indicative of chronic atrophic gastritis. Biopsy results of the prepyloric mucosa suggested mild chronic inflammation with severe intestinal metaplasia. The colonoscopy revealed a 0.6 cm wide basal raised lesion near the hepatic flexure, a 0.3 cm erosive lesion in the middle section of the transverse colon, and a 0.3 cm semipedunculated polyp in the sigmoid colon (**Figure 1**). Pathological findings indicated that the polyp in the sigmoid colon was a hyperplastic polyp, while the polyps in the transverse colon were tubular adenomas. All lesions were removed through cold snare polypectomy or submucosal resection. Four months after the gastrointestinal endoscopy examinations, the patient’s CEA level continued to increase progressively, which reached 18.5 ng/mL in July 2023 (**Table 1**).

**Table 1:** CEA levels of the patient.

Date	CEA level (ng/mL)
October, 2022	7.5
February, 2023	8.1
July 4, 2023	18.5
August 18, 2023	20.0
October, 2023	31.5
November 21, 2023	37.2
April 24, 2024	26.2
July 22, 2024	15.4
October 14, 2024	8.6
November 15, 2024	6.3
February 26, 2025	3.7
April 21, 2025	2.7
September 8, 2025	1.8

Reference range of CEA ≤5.00.

**Figure 2:**
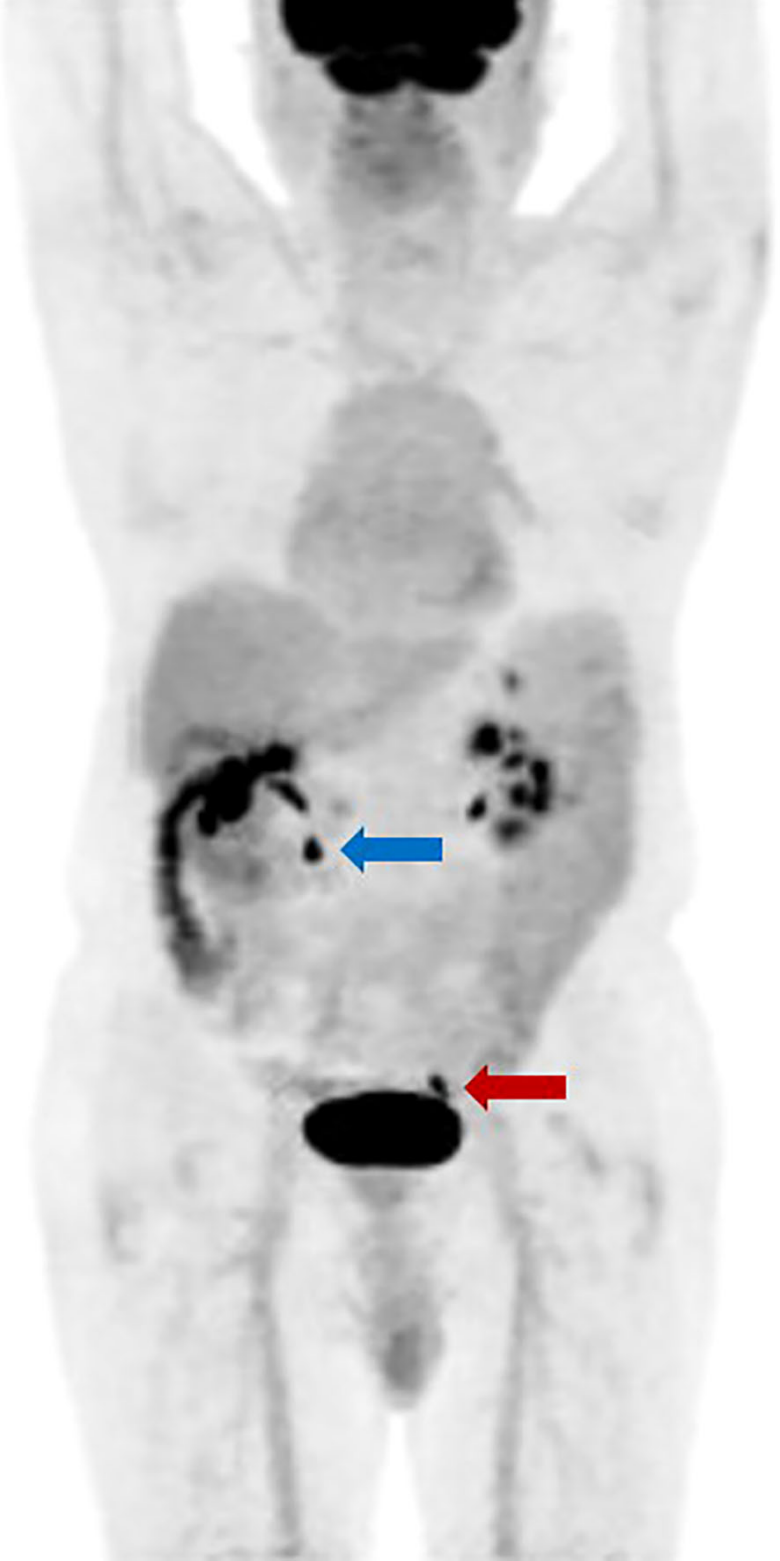
PET/CT scanning showing increased metabolic activity in the prostate. The patient exhibits slightly increased metabolic activity in the posterior prostate, with calcification spots noted (indicated by a red arrow). The ascending colon shows likely physiological uptake. There is localized increased metabolic activity in the transverse colon (indicated by a blue arrow), suggesting either physiological uptake or the presence of a small adenoma.

Considering the increasing CEA level, the patient underwent a positron Emission Tomography/Computed Tomography (PET/CT), which revealed segmental uptake in the ascending and transverse colon (SUV max 5.7). Patchy areas of decreased density in the posterior part of the prostate were also found, with slightly increased uptake (SUV max 2.8) (**Figure 2**). However, the PSA levels of the patient were within the reference range (**Table 2**) and these findings did not clearly explain the progressive increase in the patient’s CEA level. During October to November 2023, with the continuous elevation of CEA up to 37.2 ng/ml, the patient underwent low-dose chest CT, an upright abdominal X-ray, a thyroid ultrasound examination, and a small bowel endoscopy, all of which revealed no significant abnormalities. Upon re-examination with a colonoscopy under general anesthesia, the patient was found to have a 0.6 cm flat polyp in the sigmoid colon and a 2 cm broad-based, relatively flat polyp located 2 cm from the anus. Submucosal resection was performed. Pathological analysis indicated that the sigmoid colon polyp was a hyperplastic polyp, while the rectal polyp was a tubular adenoma without evidence of dysplasia. The follow-up PET/CT showed no significant changes compared to the previous results. The CEA level of the patient still remained elevated until November 21, suggesting the need for further exploration.

**Table 2:** PSA levels of the patient.

Date	PSA-T level (ng/mL)	PSA-F level (ng/ml)	F/T
August 18, 2023	2.710	0.575	0.21
October 20, 2023	3.260	0.743	0.23
November 20, 2023	2.760	0.696	0.25
December 28, 2023	3.110	0.733	0.24
February 2, 2024	8.290	/	/
April 24, 2024	0.086	/	/
July 22, 2024	0.006	/	/
October 14, 2024	<0.006	/	/
February 26, 2025	<0.006	/	/
April 21, 2025	<0.006	/	/
September 8, 2025	<0.006	/	/

Reference range of PSA-T ≤4.000.

Reference range of PSA-F ≤0.930.

**Figure 3:**
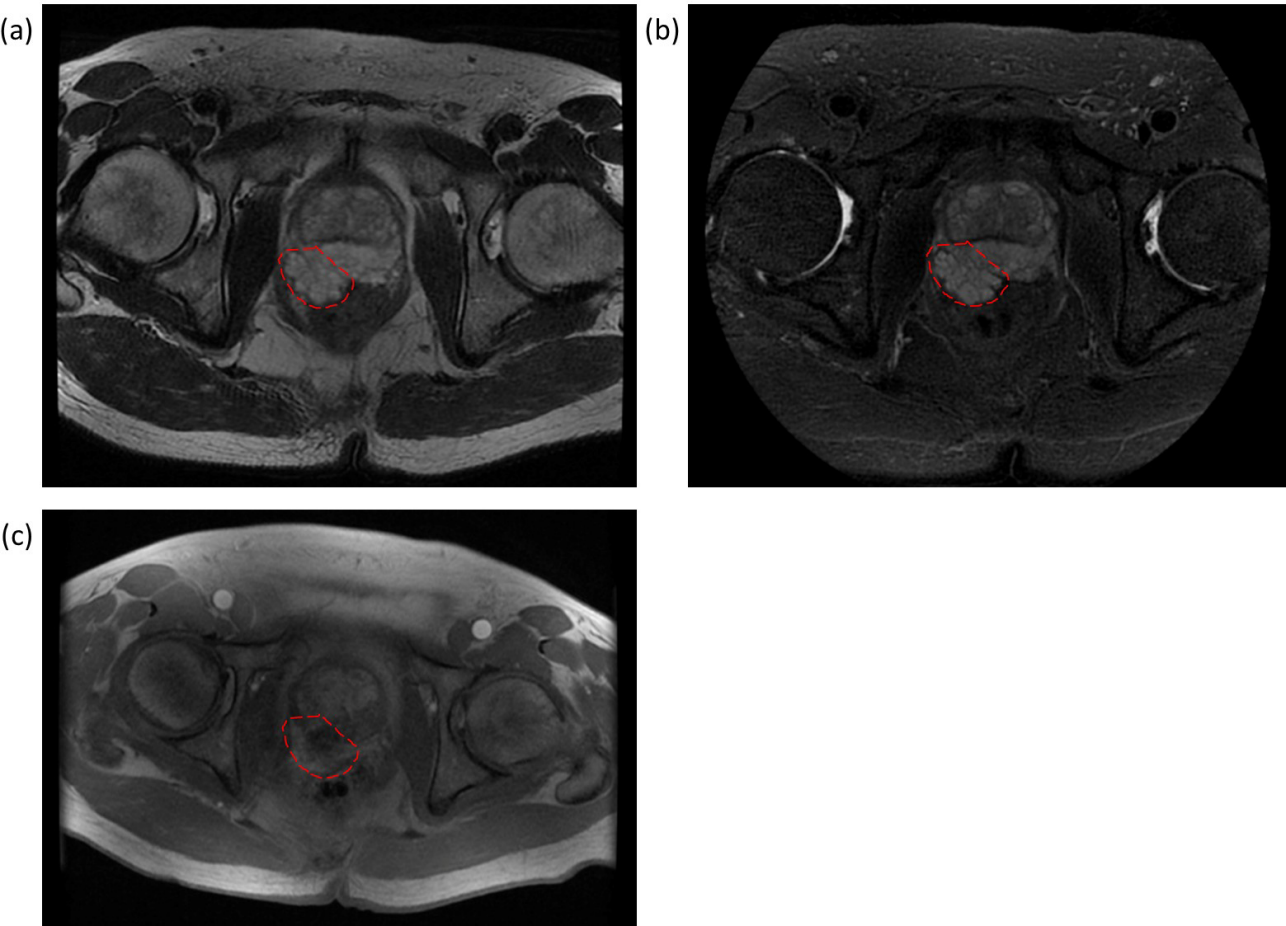
Pelvic MRI of enlarged prostate with dorsal irregular mass. The prostate is enlarged with a local protrusion towards the bladder (6 cm×5 cm×6 cm). An irregular mass is observed on the dorsal side of the prostate, closely associated with the peripheral zone, with lobulated margins and poorly defined borders adjacent to the anterior rectal wall. Red dashed circles indicate the approximate location of the lesion. **(a)** Axial T2-weighted image: the lesion appears hyperintense. **(b)** Axial fat-suppressed T2-weighted image: the lesion remains hyperintense. **(c)** Axial contrast-enhanced T1-weighted image: the lesion demonstrates heterogeneous enhancement.

On December 22, 2023, unexpectedly, the results of a routine pelvic MRI with contrast enhancement of the patient showed notable abnormalities. An irregular mass shadow on the dorsal side of the prostate was found, closely related to the peripheral zone of the prostate, with a lobulated margin (**Figure 3**). It showed isointense signal on T1, high signal on T2 and T2 fat-suppressed images, significant heterogeneous enhancement at the margins on contrast-enhanced scans, and no obvious increase in DWI. The lesion is adjacent to the anterior wall of the rectum with unclear local demarcation, suggesting the possibility of an exophytic prostate tumor, and malignancy cannot be ruled out. Digital rectal examination could find a hard mass in the middle part of the prostate.

Histopathological examination of the colonic lesions obtained by endoscopic resection demonstrated typical features of benign and premalignant colorectal lesions. On hematoxylin and eosin (H&E) staining, both the sigmoid colon lesions showed features consistent with a hyperplastic polyp, while the transverse colonic and rectal lesions demonstrated tubular adenomas characterized by glandular crowding and nuclear pseudo stratification without evidence of high-grade dysplasia or invasive growth (**Supplementary Figure 1**). No extracellular mucin pools, signet-ring cells, or infiltrative architecture were identified. Based on these findings, there was no histopathological evidence suggestive of malignancy of gastrointestinal origin.

**Figure 4:**
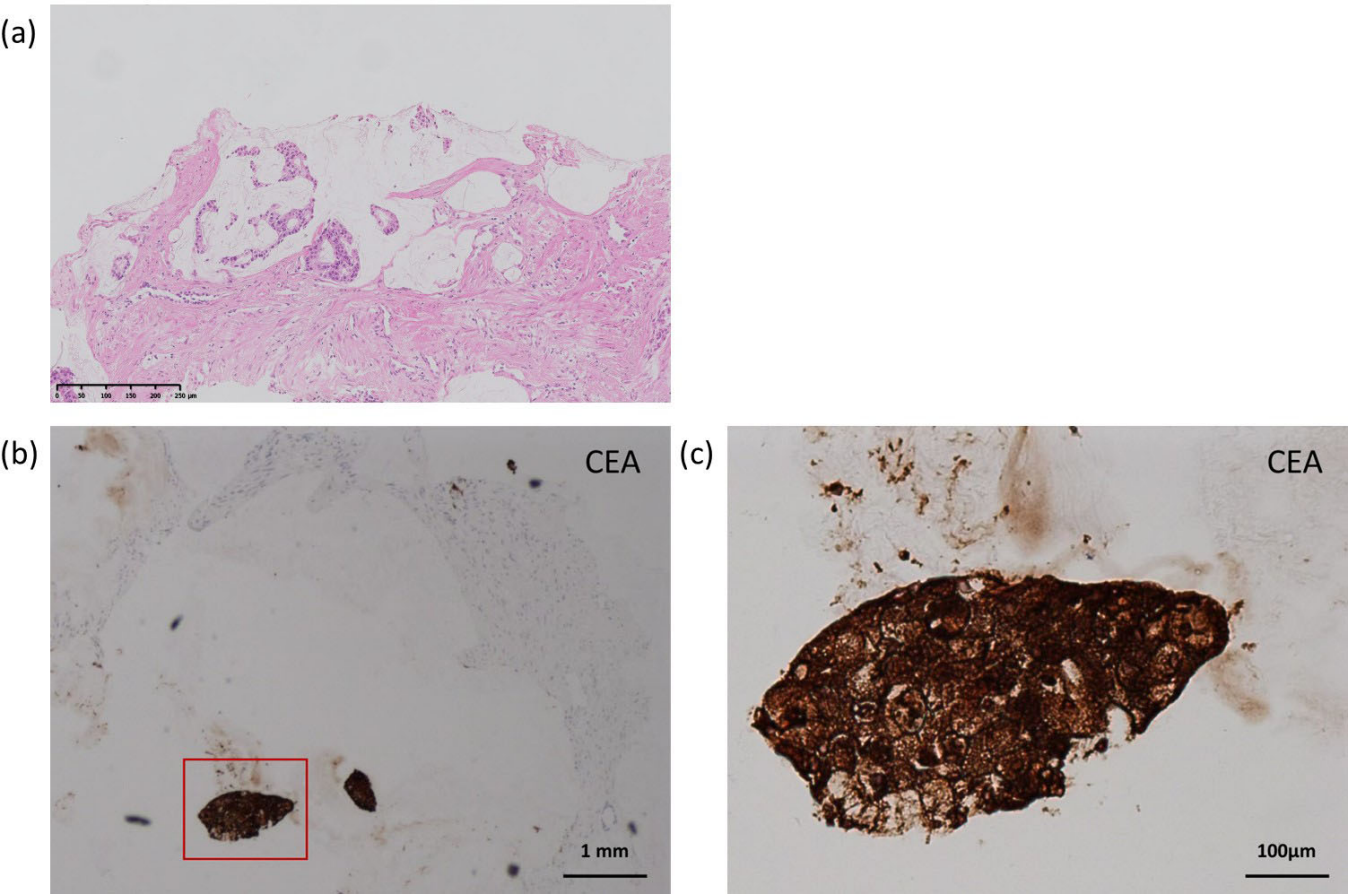
Histopathology and CEA immunohistochemistry of prostatic lesion. **(a)** H&E staining showing adenocarcinoma with mucinous features (×10). **(b)** CEA immunostaining at low magnification (×4); red box indicates the region in **(c)**. **(c)** CEA immunostaining at high magnification showing strong cytoplasmic positivity (×20).

Given the persistent and progressive elevation of serum carcinoembryonic antigen (CEA), further evaluation of the prostate was pursued. Prostate core needle biopsy specimens revealed infiltrative atypical glands with abundant extracellular mucin on H&E staining, consistent with prostate adenocarcinoma with mucinous features (**Figure 4**). The tumor was graded as Gleason score 8 (4 + 4).

Immunohistochemical analysis of the prostatic lesion showed positivity for P504S, androgen receptor (AR), and NKX3.1, supporting prostatic origin. The tumor cells were negative for PSA, chromogranin A, synaptophysin (except for focal weak positivity), SATB2, GATA3, and PAX8. Notably, strong cytoplasmic staining for CEA was observed in malignant cells, whereas surrounding stromal tissue was negative (**Figure 4**). This immunophenotype was consistent with a primary prostatic adenocarcinoma with mucinous features and provided a pathological explanation for the elevated serum CEA levels.

It should be emphasized that a definitive diagnosis of primary mucinous (colloid) adenocarcinoma of the prostate requires demonstration of more than 25% extracellular mucin within the tumor and can only be established on prostatectomy specimens. As the current diagnosis was based on core needle biopsy, the lesion was appropriately classified as prostate adenocarcinoma with mucinous features rather than primary mucinous adenocarcinoma.

### Follow-up and outcomes

Following the pathological diagnosis, the patient underwent definitive radiotherapy consisting of 60 Gy delivered in 20 fractions between January 26 and February 21, 2024. Androgen deprivation therapy with goserelin was initiated concurrently. Subsequently, localized dose-escalation radiotherapy was administered between May 27 and June 3, 2024. During follow-up, serum CEA levels showed a gradual decline, while PSA levels remained within the normal range without biochemical evidence of disease progression. At the most recent follow-up, imaging and laboratory findings indicated satisfactory local disease control, and the patient remained clinically stable under continued surveillance. A timeline summarizing the diagnostic workup and treatment course is presented in **Supplementary Figure 2**.

## Discussion

Carcinoembryonic antigen (CEA) serves as a crucial auxiliary diagnostic marker for colorectal carcinoma. Elevated CEA levels often prompt the suspicion of colorectal cancer. However, CEA elevation can also be seen in other malignancies, including medullary thyroid carcinoma, pancreatic cancer, hepatocellular carcinoma, and prostatic carcinoma. Given the limited specificity and sensitivity of CEA, it is typically essential to evaluate it alongside other tumor markers. Prostate-specific antigen (PSA) is a reliable biomarker for prostatic carcinoma screening. For patients suspected of prostatic carcinoma without imaging features and PSA elevation, follow-up testing of PSA is recommended (6). A cohort study also found elevation of CEA in a certain proportion of patients with androgen-independent prostatic carcinoma (7). Two cell types of primary prostate adenocarcinoma were identified, which favor different paths of metastasis (8). The CEA-producing cancers selectively metastasize to the liver while the PSA-producing cancers tend to spread to lymph nodes. However, patients of primary prostatic carcinoma with uniquely elevated CEA and normal PSA were rarely reported (8). The individual in this case report was a prostatic carcinoma patient with an initial presentation of elevated CEA levels and normal PSA levels. After the detection of elevated CEA, he underwent meticulous follow-up and comprehensive systemic examination over several months, which contributed to an earlier diagnosis. PET/CT showed a decrease in the posterior prostate density and a slight increase in metabolism, suggesting malignant lesions for the first time. However, the normal PSA level and the history of benign prostatic hyperplasia hindered the judgment of malignance. Pelvic MRI with contrast reveals suspicious tumor lesions and provides an indication for prostate needle biopsy. The biopsy results established that the elevated serum CEA levels were attributable to the primary prostatic carcinoma lesion. Because definitive classification of mucinous adenocarcinoma requires evaluation of prostatectomy specimens, the present diagnosis based on core needle biopsy was conservatively rendered as prostate adenocarcinoma with mucinous features. This suggests that CEA can potentially contribute to the early diagnosis of prostatic carcinoma independently if followed by proper imaging examination (9).

This case highlights several important diagnostic challenges. Persistent elevation of CEA in the absence of gastrointestinal malignancy often prompts extensive digestive tract evaluation, which may delay identification of extra-gastrointestinal primary tumors. Although prostate-specific antigen (PSA) remains the cornerstone biomarker for prostate cancer screening, a subset of prostatic carcinomas may demonstrate normal PSA levels, particularly in cases with mucinous differentiation or altered secretory profiles. In such settings, reliance on PSA alone may be insufficient.

Several limitations of this case should be acknowledged. First, histopathological assessment was limited by the use of biopsy specimens rather than prostatectomy material. Second, as a single case report, generalizability is limited. Nonetheless, this case underscores the potential diagnostic value of unexplained CEA elevation in elderly male patients and suggests that prostate imaging, particularly multiparametric MRI, should be considered even in the presence of normal PSA levels when conventional evaluations fail to identify a source.

CEA levels over 10 ng/mL or continuous uptrend are commonly considered as associated with malignant conditions. For individuals without CRC history, a PET/CT or whole-body CT are recommended to look for malignancy (10). Organ-specific testing might be more efficient based on the results of PET/CT. For elderly male patients with a *de novo* raised CEA, prostatic carcinoma should be specially considered. In view of the relatively high cost of PET/CT, MRI might also be an appropriate test for prostate examination. Until the underlying cause is found, the CEA level needs to be closely monitored, and repeated investigation is necessary.

### Patient perspective

When I first learned about the elevated CEA level from my routine check-up, I looked it up online and found it was often linked to gastrointestinal tumors. Naturally, my first thought was to visit the gastroenterology department. However, after undergoing both gastroscopy and colonoscopy, nothing significant was found. Later, a PET scan did show some abnormality in the prostate, but it didn’t raise much concern at the time. It was only when I had the MRI that the prostate tumor was clearly identified. The doctors told me my case was quite uncommon and that it was detected relatively early. They also mentioned that if I had not been so proactive with the imaging tests, the tumor might have been missed. I am grateful to the doctors in both the gastroenterology and urology departments for enabling this early diagnosis and treatment. Now, I feel more confident about my health. I am following the treatment plan step by step and focusing on staying positive.

## Data Availability

The original contributions presented in the study are included in the article/**Supplementary Material**. Further inquiries can be directed to the corresponding author.

## Supplementary material

Supplementary Figure 1 H&E-stained sections of colonic polyps obtained by endoscopic resection. **(a)** Transverse colon polyp from the first colonoscopy, showing tubular adenoma (×4). **(b)** Sigmoid colon polyp from the first colonoscopy, showing hyperplastic polyp (×4). **(c)** Sigmoid colon polyp from the second colonoscopy, showing hyperplastic polyp (×4). **(d)** Rectal polyp from the second colonoscopy, showing tubular adenoma without high-grade dysplasia (×4). Scale bars: 500 μm.

Supplementary Figure 2 Timeline of clinical management. Summary of the patient’s clinical course from initial CEA elevation through diagnosis and treatment. ADT, androgen deprivation therapy; CEA, carcinoembryonic antigen.
