# Supplementary figures and images for: Prostatic carcinoma with elevated carcinoembryonic antigen: a case report
Source: Front Oncol. 2026 Mar 9;16:1701428. doi: 10.3389/fonc.2026.1701428 (PMC13006275; doi:10.3389/fonc.2026.1701428)

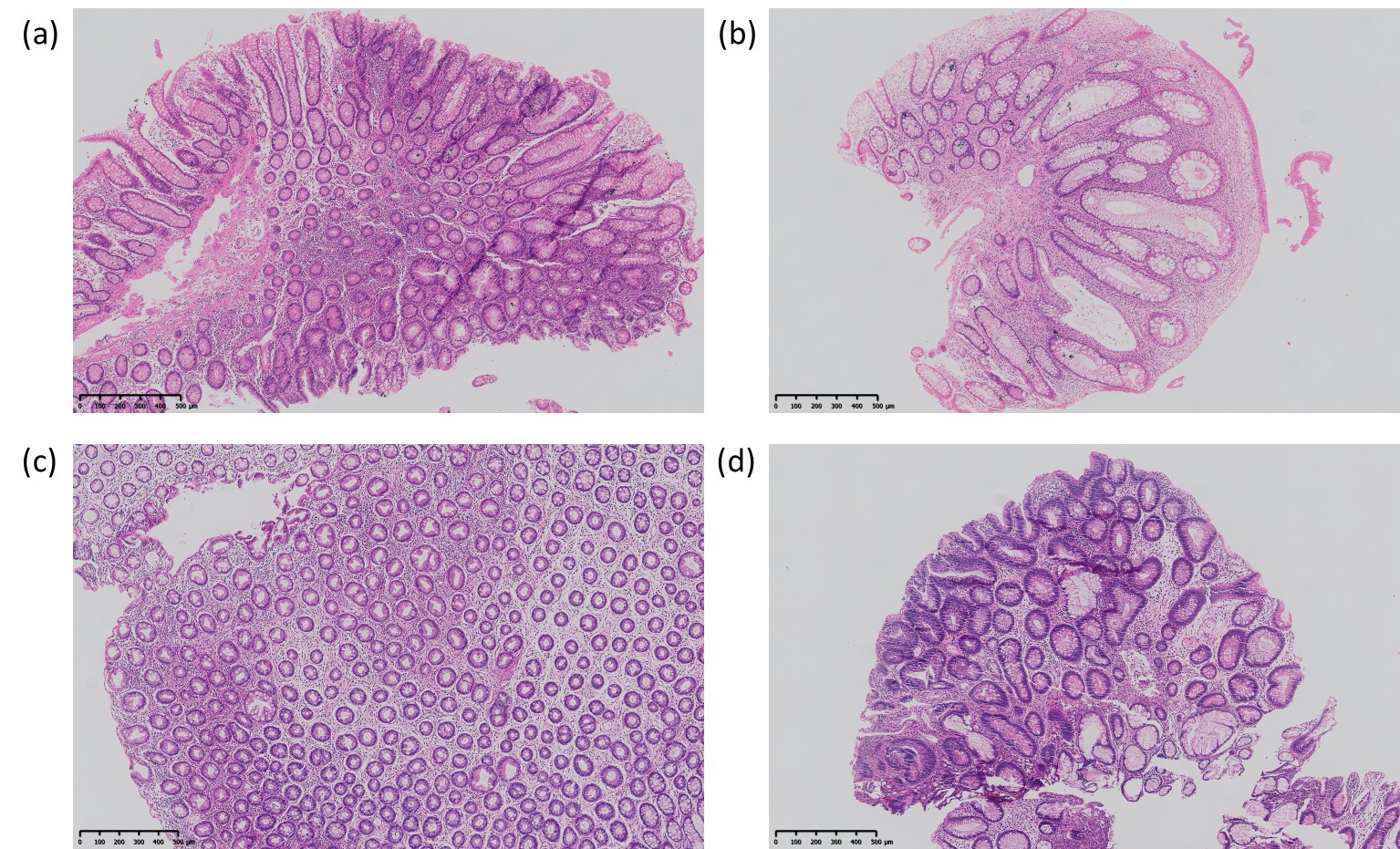

Supplement: Supplementary Figure 1 — H&E-stained sections of colonic polyps obtained by endoscopic resection. (a) Transverse colon polyp from the first colonoscopy, showing tubular adenoma (×4). (b) Sigmoid colon polyp from the first colonoscopy, showing hyperplastic polyp (×4). (c) Sigmoid colon polyp from the second colonoscopy, showing hyperplastic polyp (×4). (d) Rectal polyp from the second colonoscopy, showing tubular adenoma without high-grade dysplasia (×4). Scale bars: 500 μm. [file Image1.jpeg]

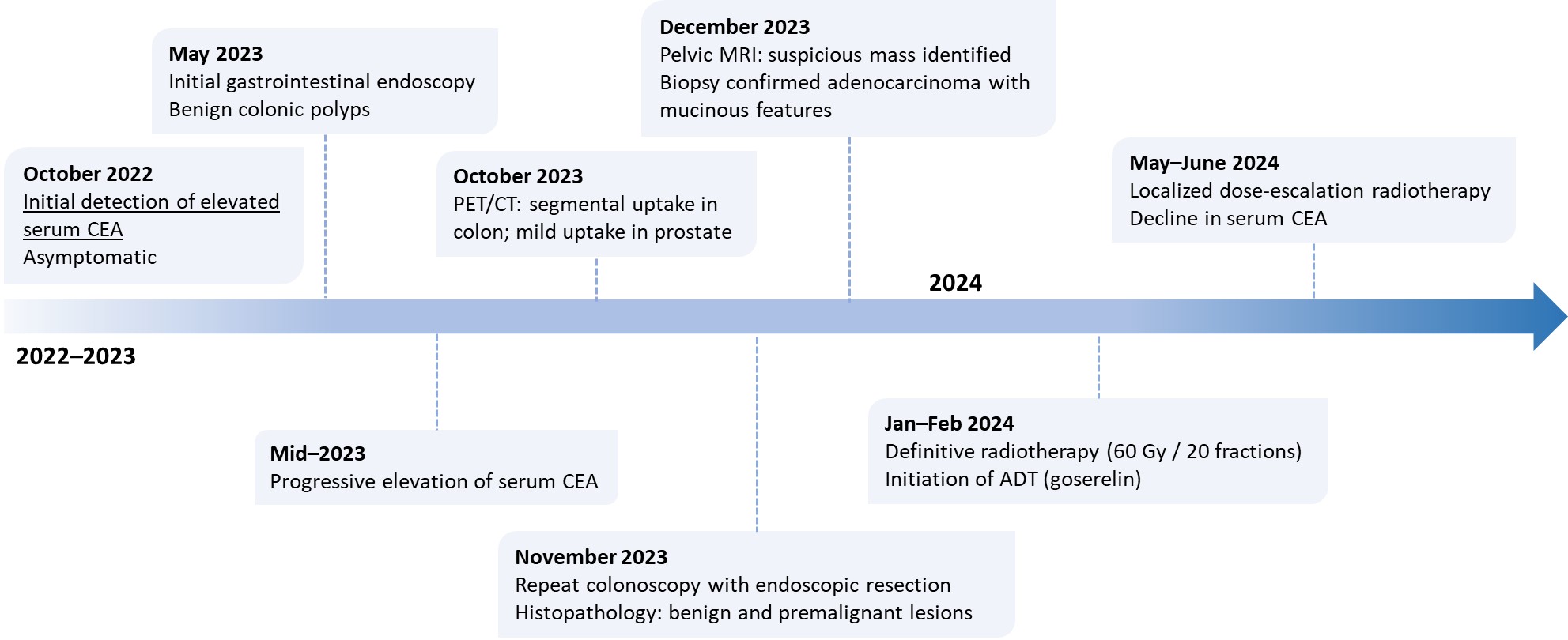

Supplement: Supplementary Figure 2 — Timeline of clinical management. Summary of the patient’s clinical course from initial CEA elevation through diagnosis and treatment. ADT, androgen deprivation therapy; CEA, carcinoembryonic antigen. [file Image2.jpeg]
